# Aging, Aerobic Activity and Interhemispheric Communication

**DOI:** 10.3390/brainsci2040634

**Published:** 2012-11-16

**Authors:** Keith M. McGregor, Kenneth M. Heilman, Joe R. Nocera, Carolynn Patten, Todd M. Manini, Bruce Crosson, Andrew J. Butler

**Affiliations:** 1 Atlanta Rehabilitation Research and Development Center of Excellence, Department of Veteran’s Affairs, Decatur, GA 30033, USA; Email: joenocera@emory.edu (J.R.N.); bruce.crosson@emory.edu (B.C.); andrewbutler@gsu.edu (A.J.B.); 2 Department of Neurology, Emory University, Decatur, GA 30033, USA; 3 Department of Veteran’s Affairs Brain Rehabilitation and Research Center, Gainesville, FL 32609, USA; Email: heilman@neurology.ufl.edu (K.M.H.); patten@phhp.ufl.edu (C.P.); 4 Department of Neurology, University of Florida, Gainesville, FL 32611, USA; 5 Department of Physical Therapy, University of Florida, Gainesville, FL 32611, USA; 6 Department of Aging and Geriatric Research, University of Florida, Gainesville, FL 32611, USA; Email: tmanini@ufl.edu; 7 Department of Physical Therapy, Georgia State University, Atlanta, GA 30303, USA

**Keywords:** aging, physical fitness, interhemispheric communication, transcranial magnetic stimulation, dexterity

## Abstract

Recent studies have shown that during unimanual motor tasks, aging adults show bilateral recruitment of primary motor cortex (M1), while younger adults show a suppression of the ipsilateral motor cortex. Additional work has indicated that increased bilateral M1 recruitment in older adults may be deleterious when performing some motor tasks. However, higher levels of physical fitness are associated with improved dexterity and fitness may mitigate the loss of both inhibitory and excitatory communication in aging adults. The goal of this study was to assess dexterity and interhemispheric motor communication in physically fit and sedentary middle-age (40–60 years) right handed participants using tests of hand deftness and transcranial magnetic stimulation (TMS). To behaviorally assess the influence of interhemispheric communication on motor performance, participants also perform the coin rotation deftness task while maintaining pinch force with the opposite hand (bimanual condition). We correlated these behavioral measures with the ipsilateral silent period using TMS to assess interhemispheric inhibition. Our results show that the middle-aged adults who were physically fit had better dexterity of their right hand (finger tapping and peg-board). When performing the coin rotation task the fit group had no between hand differences, but the sedentary group’s left hand performance was inferior to the their right hand. We found that better dexterity correlated with ipsilateral silent period duration (greater inhibition) thereby supporting the postulate that fitness improves interhemispheric motor communication.

## 1. Introduction

A decreased ability to perform upper extremity motor functions such as a loss of dexterity [1], precision grip [2], impaired motor learning [3], increased reaction time [4], and slower psychomotor speed [5] have been reported as a result of the aging process. The cause of these decrements may include impaired mechanical factors such as muscle atrophy [6], decreased density and pliability of cartilage [7], and an increase in the rigidity of other connective tissues [6]. However, in the absence of inflammatory conditions, perhaps the most significant explanation of age-related unimanual motor dysfunction resides in central nervous system changes that occur as a result of aging [8,9,10]. These changes have recently been explored using functional neuroimaging and neurophysiological techniques. Numerous studies using these methods have indicated that older age is associated with decreased levels of interhemispheric communication including both inhibition and excitation [11,12,13,14,15,16,17]. However, the effect of this age-related alteration in neural communication on motor performance remains poorly understood. 

The use of transcranial magnetic stimulation has offered insight into interhemispheric communication using both single and paired pulse magnetic stimulation to the primary motor cortex to measure the effect on target muscles. One such measure, the ipsilateral silent period, is a single pulse TMS paradigm that is relatively straightforward to implement and provides an index of interhemispheric inhibition. The ipsilateral silent period is a period of reduced muscle activity during an isometric muscle action observed following magnetic stimulation of the motor cortex ipsilateral to the active hand. The finding that the ipsilateral silent period is absent in patients with agenesis of the corpus callosum [18] indicates that the route of inhibition likely involves interhemispheric transfer, although most likely via activation of ipsilateral interneurons; see [19]. The ipsilateral silent period has been shown to decrease in duration in older adults [12,13,14,20] indicating lower levels of interhemispheric inhibition with increasing age. However, the effects of these changes in interhemispheric inhibition on motor performance are not well known. Recently, Fujiyama *et al.*, (2012) [14] reported that older adults who exhibited lower levels of interhemispheric inhibition (as assessed by the ipsilateral silent period) revealed the most difficulty with intramanual motor synchronization tasks. Additionally, McGregor *et al.*, (2011) also reported that elderly (>60 years) adults who exhibited shorted ipsilateral silent periods had somewhat slower reaction times [12]. These findings suggest that age-related decreases in interhemispheric inhibition, appears to impair motor performance. However, the alterations in motor activities induced by this decrease in interhemispheric inhibition have not been fully explored. 

Changes in interhemispheric communication may not be an inevitable consequence of aging. Physical fitness has long been associated with maintenance of neurocognitive functions [21]. There is evidence that individuals with higher levels of physical fitness may not show the same interhemispheric transfer patterns as their sedentary age matched counterparts, such that older adults who remain physically active tend to show a less severe decline in measures of interhemispheric inhibition, as compared to sedentary older adults [12]. For example, studies have revealed that older adults who remain highly physically active for over five years (contiguous to assessment) tend to show interhemispheric activity that is more similar to younger adults than to their sedentary counterparts [12,22]. Therefore, it is possible that higher levels of physical activity may confer protective effects that preserve both neurophysiological functioning and corresponding motor performance. However, the effects of this preservation of interhemispheric communication have not been fully studied in the context of motor performance in aging adults.

Most aging studies have focused on age extremes (<30 years *vs.* >65 years). Although using extremes this practice has been successful in gaining an understanding how aging changes the motor system, studies of participants in the intermediate range of ages (40–60 years) may improve our understanding of the physiological process of aging. Those few studies that have included middle-age adults have reported changes in the motor system between ages 40–60 years [23,24]. Given the significant incidence of motor related disorders in middle-aged adults, the paucity of motor studies in this cohort is surprising [25,26] and accounts for a considerable gap that exists in our understanding regarding the onset and remediation of age-related motor dysfunction. As such, we chose to enroll participants between the ages of 40 and 60 for the present study.

The goal of this study was to test the hypothesis that middle-age individuals with greater aerobic fitness would demonstrate superior upper extremity dexterity than those who are less fit and this greater dexterity would be associated with higher levels of interhemispheric communication, both inhibition and excitation. Thus in this study we investigated and compared the relationship between upper extremity motor dexterity, reaction time and interhemispheric inhibition in 40 middle aged right handed participants who were either highly physically fit or less physically fit, as determined by self report and behavioral assessment. We tested interhemispheric inhibition by evaluating the ipsilateral silent period (iSP), which increases with increased inhibition. The iSP was measured by single-pulse transcranial magnetic stimulation (TMS). Dexterity was measured using the nine-hole pegboard task and a coin rotation task. To test the effect of interhemispheric activity on motor function, participants performed the coin rotation task in two conditions. In the unimanual condition, participants rotated a coin twenty times as rapidly as possible with each hand. In the bimanual condition, the participant performed isometric pinch with either the right or left hand, while performing the coin rotation task with the opposite (target) hand.

## 2. Methods

### 2.1. Participants

Of the 190 candidates screened by telephone, 40 right-handed middle-age (ages 40–60) individuals participated. At the time of screening and study participation, all participants were reportedly healthy and without cardiovascular diseases (*i.e.*, angina, myocardial infarction, cardiac arrest, or uncontrolled hypertension), as well as being free of metabolic, neurological, psychological or musculoskeletal disorders. Based on self-reported exercise activity on the Physical Activity Readiness Questionnaire (PARQ) [27], qualified participants were identified into one of two categories, relatively sedentary or physically active. Aerobic fitness was later tested via a direct test of estimated value of maximal volume of oxygen uptake (VO_2max_). Two participants expressed discomfort during the experimental procedure and elected to withdraw from participation. The relatively sedentary group (*n* = 21; 12 female) of middle-age adults (heretofore: sedentary) was comprised of healthy individuals that engaged in voluntary cardiovascular exercise for fewer than 45 total minutes per week, as determined by the Leisure Time Activity Questionnaire (LTAQ) [28] and Physical Activity Readiness Questionnaire (PARQ). The physically fit and active group (heretofore: physically fit) of middle-age adults (*n* = 17; 8 female) was comprised of healthy individuals who reportedly engaged in bouts of voluntary aerobic (swimming, bicycling, jogging, *etc.*) exercise lasting at least 45 min at least three times per week. As we were interested in long-term effects of chronic exercise, to be enrolled these physically active adults were required to have engaged in these active exercise patterns for at least five consecutive years immediately preceding the current study. The strength of right-hand dominance was equivalent between groups as assessed by the Edinburgh Handedness Inventory [29]. 

Participants were also screened for compatibility with transcranial magnetic stimulation (TMS) and were excluded if they reported metal implants above the waist, history of seizure, repeated migraine headaches or history of Meniere’s disease. Participants on beta-blocking hypertensive medications were also excluded due to effects on heart rate during estimated VO_2max_ testing. Finally, participants who reported significant skilled hand practice (musical instruments, typists, expert needlepoint, *etc.*) were excluded from participation if they engaged in the preferred activity for longer than three 1-h sessions per week. Study personnel completed the informed consent process with each participant following protocols approved by the University of Florida’s Institutional Review Board (IRB) and the Malcom Randall VA Medical Center Research and Development Committee. Participant characteristics are listed in Table 1.

**Table 1:** Participant characteristics—Mean ± SD (range).

	Active Middle-Age	Sedentary Middle-Age
Age	51.3 ± 5.9 (41–60)	52.6 ± 6.9 (41–60)
*N*/Gender	17/8 Female	21/12 Female
Education	16.1 ± 2.25 (12–20)	16.4 ± 2.38 (12–20)
BMI *	22.1 ± 2.5 * (18–25)	26.7 ± 3.9 * (22–32)
VO_2max_ *	49.8 ± 12.7 * (36–69)	29.6 ± 6.6 * (20–37)
Weekly Activity (min/bout) *	146 ± 23.4 * (45–180)	36.7 ± 12.5 * (15–45)

BMI: Body Mass Index; Cell values denote group means; Parentheses indicate standard deviation within cell; Bracket indicates range; * Student’s *t*-test contrast significance at *p* < 0.05.

### 2.2. Assessments

Upper extremity motor function and physical activity were measured at an initial behavioral testing session. Basic physiological metrics (height, weight) and vital signs (resting heart rate, blood pressure) were also taken at this session. Participants returned at least two days later for a second session during which TMS measures were obtained while performing dexterity tasks.

#### 2.2.1. Motor Assessments

Participants completed motor assessments of the dominant hand including: grip strength, the Halstead Finger Tapping task, simple reaction time, and the Nine-Hole Pegboard task [30]. Additionally, to test distal motor dexterity, participants engaged in a coin rotation task with two conditions. In the first condition, the participant rotated a coin (U.S. nickel) twenty times as quickly as possible using the index finger, middle finger and thumb with time to completion as the outcome measure. This test is used for assessment in routine neurological practice and has been shown to be diagnostic of distal motor function both in cases of suspected pathology and aging in the absence of pathology [31,32]. In the second condition, the participant maintained an isometric pinch force of 25%–35% of maximum voluntary force with a pinch grip dynamometer using a lateral grip. Coin rotation tasks were performed with both the left and right hands. Both the hand used for coin rotation and trial condition (unimanual or bimanual task) were pseudo-randomized and counter-balanced across participants to account for potential order effects. Accidental coin drops were excluded from consideration and the participant repeated the trial if a drop occurred. Participants were allowed 3–5 practice trials to acclimate to the rotation task in each task condition. The difference score between the bimanual and unimanual task conditions was calculated to assess the effect of bimanual activity. Grip force was measured in lateral pinch, precision pinch and power grip using Jamar brand dynamometers. A maximum of ten trials are administered. The average number of taps is the outcome measure. For the simple reaction time test, E-Prime software (PST Software, Pittsburgh, PA, USA) was used to present a target stimulus on a computer screen at pseudorandomly timed intervals (250–1000 ms). The participant maintained surface contact with the right hand and depressed a computer keypad button as quickly as possible in response to the visual stimulus. 

#### 2.2.2. Estimated VO_2max_

To assess aerobic fitness level, we employed the cycle-based YMCA submaximal VO_2_ exercise assessment. This test is an accurate method to assess physical fitness level without placing maximal strain on the participant [33]. In the YMCA test heart rate workload values are obtained at 2–4 points and extrapolated to predict workload at the estimated maximum heart rate (MHR) (e.g., 220-age). VO_2max_ is then calculated from the predicted maximum workload. Participants rode a stationary bicycle for 4 three-minute stages (Table 2). The first stage was a warm-up at 50 revolutions per minute (RPM) at a power level of 25 watts. During all testing stages, heart rate was continuously monitored to ensure the participant did not exceed 85% of age-predicted maximum heart rate, at which point the exam would be stopped. For the analysis, average heart rate during the final 30 s of the 2nd and 3rd min was plotted against workload for each stage. Three-minute trial workloads, shown in Table 2 below were chosen based on the participants’ heart rate at the end of the warm-up period. The fourth 3-min stage was a cool-down period added to the end of the test. Outcome measures for the YMCA test were estimated VO_2max_ and estimated liters of oxygen consumed per minute.

**Table 2:** YMCA sub-maximal cycling protocol.

	<80 bpm *	80–90 bpm *	90–100 bpm *	>100 bpm *
Stage 1	125 watts	100 watts	75 watts	50 watts
Stage 2	150 watts	125 watts	100 watts	75 watts

* Beats per minute.

#### 2.2.3. Single-Pulse Transcranial Magnetic Stimulation

For the TMS procedure, participants sat in a comfortable chair within a stereotactic positioning frame. Electromyography (EMG) was recorded from the first dorsal interosseous (FDI) muscle on both hands. Muscle activation was monitored with a real-time oscilloscope software package (Scope 4.0, ADInstruments Ltd., Colorado Springs, CO, USA). A Magstim 200^2^ magnetic stimulator (The Magstim Company Ltd., Carmarthenshire, UK) with a 70 mm figure-of-8 coil was used to stimulate the left primary motor cortex during the initial mapping procedure. Navigation of the stimulator coil to target cortex was accomplished using coil registration to a standardized brain image provided by BrainSight 1.0 software (Rogue-Research, Montreal, Canada). During stimulation, the coil was placed tangential to the scalp with the handle pointing backwards and 45 degrees away from the midline. The scalp site (“hotspot”) corresponding to the lowest stimulator output sufficient to generate magnetic evoked potentials (MEP) of at least 50 mV in 6 out of 10 trials was defined as the area of lowest motor threshold (LMT). This hotspot was the single site of stimulation for all subsequent TMS. 

**Figure 1:**
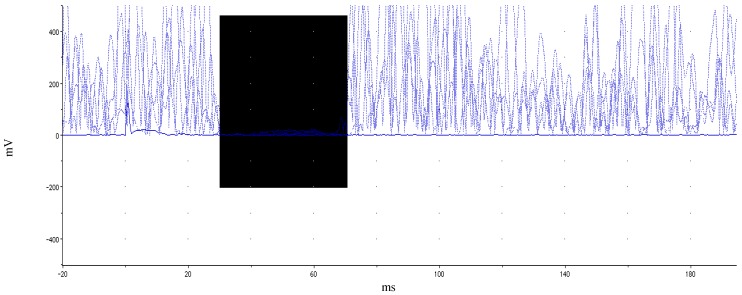
Illustration of ipsilateral silent period. Rectified EMG across multiple trials within a single participant while holding isometric force at 35%–50% MVC. Highlighted (dark) area delineates EMG depression. Motor evoked potential and subsequent silent period occurs at ~38 ms. iSP duration for this participant was 45 ms.

The TMS protocol is similar to that used in our previous work [12]. Two single-pulse TMS measures were considered for the current study: LMT value and ipsilateral silent period (iSP) duration (ms). To assess the iSP duration, the participant produced isometric pinch grip at 30%–50% maximal voluntary contraction (MVC), determined by grip dynamometer while TMS was delivered concurrently at 150% LMT to the left primary motor area FDI hotspot. EMG was monitored from the right FDI. Twenty consecutive trials were performed for the iSP assessment. After every five trials, the participant was given a brief rest to allay fatigue. During iSP assessment, the participant was instructed to maintain the non-active hand in a prone, resting position. Two TMS measures were analyzed: lowest motor threshold (LMT) and duration of ipsilateral silent period (iSP). Scope 4.0, JMP 9.0 and Microsoft Excel 2007 software were used to complete this analysis. This procedure was modified from related literature [34]. EMG data were rectified prior to analysis. EMG baseline was taken as mean of the EMG signal 20 ms pre-stimulus during pinch grip. MEP latency was measured from the onset of the stimulus presentation to the onset of the MEP. The first of five consecutive datapoints after MEP that evidenced a minimum decrease of 50% from mean EMG values from the 20 ms prestimulus recording period were taken as the silent period onset. Conversely, the first of five consecutive data points evidencing a return to greater than 50% of prestimulus mean levels identified termination of the silent period. A sample participant’s iSP is illustrated in Figure 1. Group comparisons of TMS measures were analyzed using between-subjects Student’s *t*-test and *p*-values < 0.05 were considered statistically significant.

### 2.3. Data Analysis

All data analysis was completed using Excel 2007 (Microsoft) and JMP 9 Software (SAS Institute). To test group differences on assessed variables, we used random-effects between groups *t*-tests with significance level held to *p* < 0.05. We additionally employed Pearson’s correlation analysis both within and between groups to test relationships of variables.

## 3. Results

### 3.1. Behavioral Results

Between-subjects tests of age, education, grip strength, handedness and gender did not differ between fitness groups. Table 3, below, shows the performance measures as well as between group comparisons. These comparisons revealed that the highly fit group performed better with their right hand and arm on the 9-hole pegboard and the Halstead Finger Tapping tests. Their left upper limb was not tested on these tests. However, both their right and left hands were assessed with the coin rotation task. The groups did not show any performance differences on coin rotation with the dominant (right) hand. However, the physically active group performed the rotations faster with the non-dominant hand as compared to the sedentary group. Interestingly, performance in the bimanual task did not reveal group differences on the coin rotation task for either hand. Further, when comparing isolated unimanual coin rotation during to the coin rotation in the bimanual condition the sedentary group showed a significant improvement in coin rotation performance. The physically fit group, however, did not show a significant improvement between the unimanual and bimanual conditions.

**Table 3:** Motor Performance Measures—Mean ± SD.

	Active Middle-Age	Sedentary Middle-Age
9-hole Pegboard * (s)	16.2 ± 1.76 *	18.79 ± 3.03 *
Halstead Finger Tapping * (count)	52.29 ± 7.3 *	43.82 ± 8.79 *
Simple Reaction Time (ms)	269 ± 32.25	289 ± 33.3
Coin Rotation-Right (s)	13.25 ± 2.88	14.99 ± 3.77
Coin Rotation-Left * (s)	13.05 ± 2.18 *	15.3 ± 3.3 *
Bimanual Task-Right (s)	13.16 ± 0.63	12.95 ±0.55
Bimanual Task-Left (s)	12.73 ± 1.39	12.61 ± 2.34
Task Difference-Right * (s)	0.31 ± 2.02 *	−2.74 ± 2.03 *
Task Difference-Left * (s)	0.09 ± 1.83 *	−2.03 ± 3.2 *

Task Difference: Subtraction of unimanual task time from bimanual task performance on coin rotations with the same hand; * indicates significance at *p* < 0.05 level.

### 3.2. TMS Results

**Figure 2:**
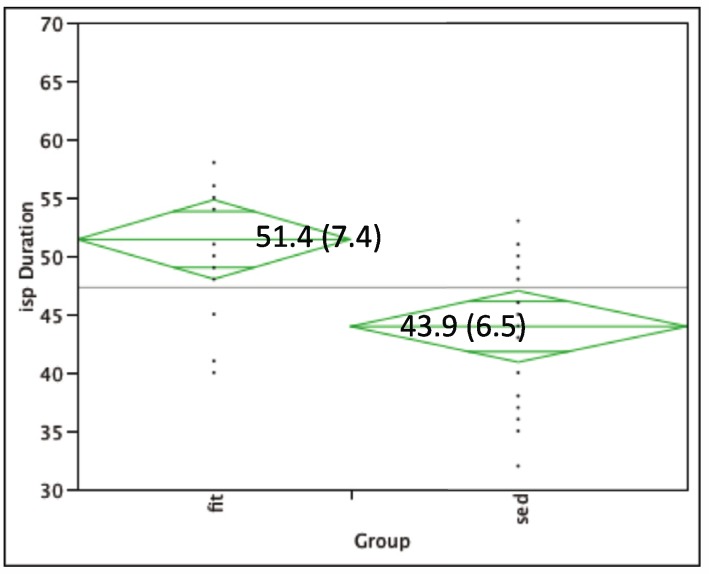
iSP duration for physically fit (left) versus sedentary groups (right). Centerline box values indicate mean (SD) of iSP duration within group.

Lowest motor threshold (LMT) was similar between groups: Fit group 44.6% (SD: 8.3); Sedentary group 45.9% (SD: 9.2). The physically fit adults (mean: 51.4 ms; SD: 7.4) showed a longer iSP duration (*p* < 0.01) as compared to sedentary adults (mean: 43.9 ms; SD: 6.4) (see Figure 2).

### 3.3. TMS and Behavioral Correlation Results

Ipsilateral silent period duration was positively correlated with VO_2max_ (*r* = 0.43, *p* = 0.01) and duration of weekly activity (*r* = 0.47, *p* < 0.01)., but negatively correlated with 9-hole pegboard task (*r* = −0.38, *p* = 0.01) and the coin rotation task-right hand (*r* = −0.36, *p* = 0.02). We also showed significant negative correlations between iSP duration and differences between unimanual and bimanual coin rotation: difference score-right hand (*r* = −0.46, *p* < 0.01), difference score-left hand (*r* = −0.38, *p* = 0.02). That is, the shorter the silent period (indicating interhemispheric inhibition), the larger the improvement during the bimanual task performance. 

## 4. Discussion

The present study yielded several noteworthy findings regarding the effects of physical fitness on motor dexterity in middle aged adults. First, highly physically fit adults reveal better performance on assessments of upper extremity dexterity (pegboard) and psychomotor tapping speed. Second, this performance correlated with the ipsilateral silent period evoked with TMS. Third, engagement of the opposite hand in a bimanual task improved motor dexterity (*i.e.*, coin rotation during bimanual conditions) in sedentary, but not fit middle-aged adults. Differences in ipsilateral silent period between physically fit and sedentary individuals are an extension of findings reported previously in elderly adults [12]. These results suggest that sustained physical activity (*i.e.*, lifetime habit) may be effective in mitigating decreases in interhemispheric motor communication and attendant declines in motor function associated with the aging process. 

While it has been known for many years, that exercise has beneficial effects on psychomotor speed [35], reaction time [36] and fine motor control [37], the underlying patterns of change in the nervous system have largely been unexplained. However, prior to exploring the role of exercise in the aging brain, it is appropriate to discuss the aging process within the sedentary brain. Only recently has research begun to explore the network dynamics within the aging brain that differ when compared to younger adults. In large part, this has been made possible by the relatively recent and rapid maturation of non-invasive brain imaging techniques. Specifically, magnetic resonance imaging and transcranial magnetic stimulation have discerned age-related changes in neural inhibition in the primary motor cortex in older adults. Studies using both fMRI and TMS have established a loss of interhemispheric inhibition (IHI) in the primary motor cortex that accompanies the aging process [11,16,17,20]. However, there has been some controversy as to whether or not this loss of inhibition of the primary cortex reflects a compensatory function, or represents a decline in motor abilities caused by a different mechanism. In this latter case, reduced IHI may simply represent a neutral marker of aging. While some studies have indicated that ipsilateral hemisphere recruitment in older adults may compensate for other changes in the nervous system and positively impact motor performance [1,38,39,40,41], other studies indicate that age-related loss of interhemispheric inhibition of the primary cortex is associated with impaired motor function [12,13,14,42,43]. Given the extreme complexity of intracortical and interhemispheric inhibition, it is possible that the tasks assessed play a large role in the variability of these findings and influence our ability to differentiate age-related differences in motor control.

We assessed motor performance with multiple measurements of hand function including psychomotor speed, reaction time, and dexterity. In our assessments, longer ipsilateral silent period duration was associated with accurate spatial positioning of objects (9-hole pegboard), and higher psychomotor speed (Halstead tapper). However, we also wanted to evaluate performance on a task that involves fine, precise and coordinated independent finger movements. Thus, to further, to assess the interhemispheric effects on distal upper extremity deft motor performance in unimanual and a bimanual task conditions we selected a coin rotation task [31,32] and for the bimanual condition we added a condition that engaged the opposite hand in producing and maintaining pinch force.

In regard to unimanual performance, for the highly fit group we did not find any significant difference between their right and left hands’ performance on the coin rotation task. In contrast, the sedentary group’s performance with their left hand was significantly poorer than the performance of their right hand. Studies of patients with right and left hemisphere strokes using the coin rotation task revealed that both patient groups had a contralesional motor deficits, but the subjects with left hemisphere damage, and not those with right hemisphere damage, also had impaired coin rotation with their ipsilesional left hand [31]. These results suggest that normally the left hemisphere helps to mediate deft movements of the left hand. This left hemisphere influence on the right hemisphere’s motor systems may occur via the corpus callosum. Support for the postulate that the left hemisphere’s influence on left hand deftness is mediated by the corpus callosum comes from observations of a patient with a callosal disconnection who had a severe decrement in his left hand deftness (coin rotation). Thus, in the current study the excellent left hand coin rotation performance of the fit subjects, versus the decrement of coin rotation in the sedentary subjects, may also be related to fit group’s better interhemispheric communication.

In the bimanual task condition, performance on the coin rotation task was unchanged in the highly fit group, but performance on the coin rotation task was substantially improved in the sedentary group. There are a few explanations for this result in light of previous work. First, it is possible that the engagement of the ipsilateral hand in the sedentary group prevented non-specific and potentially task-detrimental ipsilateral recruitment of the opposite hemisphere shown in previous work involving unimanual movement during fMRI in older adults [11,12]. Alternately, as previous studies have shown that the engagement of the opposite hand enhances silent period on the ipsilateral side [44], the physically fit group may have achieved a ceiling level of inhibition while enhanced interhemispheric inhibition improved motor performance in the sedentary group. An additional study involving modulation of tasks during ipsilateral silent period assessment and fMRI across fitness levels would elaborate our understanding of these results. 

While the presence of transcallosal inhibition during unimanual movements has been established, its function remains a topic of active debate. A predominant explanation of the function of this communication is the suppression of mirror movements mediated by the opposite hemisphere [44,45]. Support for this premise is derived from studies in development and motor pathology. In pre-teen children, the existence of mirror movements during both relatively simple and more complicated unimanual motor tasks has been reported for some time [46]. About the age at which the corpus callosum becomes fully myelinated, these mirror movements decrease in prevalence and a clear pattern of ipsilateral inhibition becomes evident during unimanual control [46]. In cases of motor pathology such as Kallman’s Syndrome [47,48], mirror dystonia [49], or Klippel-Feil [50] syndrome this transcallosal inhibition is altered or absent resulting in aberrant synkinesis and functional motor impairment. While more prevalent than in younger adults [38], this extreme outcome is rare in otherwise healthy older adults. However, the interaction of interhemispheric inhibition and excitation in unimanual motor performance is highly complex and thus additional study is required. 

The main goal of this research was to learn the influence of physical fitness on motor control with a focus on the effects of transcallosal communication. To examine the effects of physical fitness on motor performance we compared a group of highly fit middle-aged adults to sedentary age counterparts. As described above, it appears that the regular participation in aerobic activity offers prophylaxis against age-related loss of interhemispheric communication. The neurochemical mechanism of action by which aerobic exercise exerts these effects is currently unknown and offers an important opportunity for translational research. Exercise is known to affect levels of brain derived neurotrophic factor (BDNF), which increases after aerobic activity and can be detected in serum blood draw [51,52]. Unfortunately, the current study did not assess levels of BDNF across groups, and, as such, we cannot make inferences as to its effects in a cross-sectional model. Future studies are necessary to measure both serum level of BDNF, genetic presentation (polymorphisms exist that show differential effects), and serum level response to focal exercise activity. However, in addition to BDNF, other mechanisms that may be responsible for the positive effects of exercise have been offered. It is possible that muscle myokines released during exercise may offer an anti-inflammatory effect on nervous system tissue that improves neural function [53] by diffusing oxidative effects of fatty acid metabolism. Additionally, the cerebrovascular system’s capillary network receives great benefit by regular exercise, perhaps through increases in vascular endothelial growth factor with improved efficiency of oxygen transport to neural tissue [54,55]. Given these potential biochemical changes, it is clear that additional research involving neurochemical markers and genetic polymorphisms be integrated into future research involving the effects of exercise on the aging nervous system. It would also be interesting to correlate these motor systems measures with measures of executive function and cognition that have been shown to change with increased chronic physical activity [56].

We would like to address some notable improvements that could be made to studies similar to the present work. First, the use of a cross-sectional design is somewhat limited by the nature of the inquiry as individual variance over time cannot be captured. Future work in this arena should focus on intervention models with multiple assessment time periods to capture individual variance inherent with behavioral studies of aerobic activity (see [56,57,58]). Use of a cross-sectional design with highly selective inclusion criteria can somewhat bias sampling. Our choice of physical activity duration for group assignment does not properly represent the influence of genetic factors that can affect motor performance and physical fitness level. Further, duration of activity potentially introduces lifestyle factors that may not be representative of a generalized population. For example, in the present example, a number of individuals from our physically fit group engaged in physical activity as part of their profession (*n* = 8) (e.g., personal trainers or semi-professional athletes). As a result, the assessed values of physical fitness and reported overall weekly exercise activity are well above the normative values (and even ACSM recommendations) for physical activity in this age group. This was an intended outcome as we strived to study a clear stratification between the fitness levels of the two groups from the outset of the study. While we acknowledge that this may somewhat limit how accurately we can infer these findings to the general population, our present results indicate the strong effect of physical activity level on the motor system of middle aged adults and far outweigh the potential limitations. Finally, the mechanism by which exercise enhances connectivity is not directly addressed by the current study. Studies using serum blood draw to evaluate exercise induced change in levels of brain-derived neurotrophic factor (BDNF) have indicated that this endogenous compound can have strong effects on neurogenesis and synaptic function [51]. Future work should endeavor to involve assay-based assessment of level of neurotrophic factors via blood draw to implicate the neurochemical mechanism of change.

## 5. Conclusions

In summary, the current work shows that, in middle age adults, higher levels of interhemispheric inhibition may be related to better motor function during unimanual tasks. Further, increased levels of physical fitness may be associated with increased interhemispheric inhibition and improved motor performance as we age. Finally, in sedentary groups, motor performance improved with the execution of a bimanual task in comparison to unimanual task conditions. This may indicate that bilateral motor cortex recruitment during unimanual tasks interferes with performance.
